# Impact of perceived social support and psychological capital on university students’ academic success: testing the role of academic adjustment as a moderator

**DOI:** 10.1186/s40359-023-01385-y

**Published:** 2023-10-17

**Authors:** Mehdi Hassan, Shuanghu Fang, Aamir Abbas Malik, Tauqeer Ahmad Lak, Muhammad Rizwan

**Affiliations:** 1 School of Educational Science, Anhui Normal University, Wuhu, China; 2 College of Public Administration, Nanjing Agricultural University, Nanjing, China; 3 Department of Sociology & Criminology, University of Sargodha, Sargodha, Pakistan; 4 School of Education Science, Nanjing Normal University, Nanjing, China

**Keywords:** Psychological capital, Perceived social support, Academic adjustment, Academic success

## Abstract

**Background:**

Academic adjustment is a significant predictor of the academic success of students. The aim of this study is to examine how academic adjustment plays an important role as a moderator in perceived social support, psychological capital, and success outcome relationships among university students.

**Methods:**

Three hundred seventy-three valid questionnaires were collected from different departments of different universities using convenience sampling method. Smart PLS 3.0 was used for data analysis.

**Results:**

The study results indicated that perceived social support and psychological capital have a significant direct impact on academic adjustment and academic success. The results of the study also demonstrated that the relationships between perceived social support, psychological capital, and successful outcomes are partially and moderated by academic adjustment.

**Conclusion:**

This research develops a predictive model for examining students’ academic adjustment to university and the outcomes of success based on social capital theory and conservation of resources theory. The current study suggests that it is necessary for policymakers to make full use of their ability to enable students to adjust to university life effectively. Higher education institutions should therefore pay full attention to the development of students’ academic skills that contribute to academic success.

## Introduction

Students at the age of university level, face crucial periods when developing themselves psychologically, professionally, physiologically, and socially. These multidimensional changes influence their well-being, university adjustment, and adaptation. Adult students are found to be inspirational, competitive, experimental, and vigour in universities [1, 2]. This phenomenon regarding students’ academic achievements, personal fulfilment, behavioural, and psychological aspects gained much attention at the global level [3, 4] As universities play an important role in achieving overall development. The university period is very critical to the development and success of the lives of young people and prepares them for the future [5]. In a university setting, academic success is one of the most appropriate outcomes, which refers to information that students gain from degree programs, whereas excellence refers to a higher level of technical, practical, and theoretical knowledge [6]. Individuals’ career success and employability can be increased through academic performance and learning. However, the process of university education carries diversity in terms of students’ individual differences, and socioeconomic backgrounds, often vary considerably in terms of prior education, basic potential, socio-emotional needs, and motivation. Some students enter universities with psychological and cognitive resources that support them effectively and enable them to meet their challenges. While others find it difficult to adjust to the requirements and complexities of the educational setting; thus, they are highly susceptible to anxiety and stress [7, 8].

Academic adjustment and experience is complex because of conflicts and significant changes related to university environment. It associated with academic success, graduate attributes, motivation, and independency directly and indirectly, Academic adjustment is a multidimensional construct that is assessed in four areas of student functioning: social adjustment, institutional adjustment, academic achievement and personal emotional adjustment [9, 10]. These integrations in university environment play vital role for students’ academic success and lifelong adjustment. It is an important measure that shows how the student has adjusted to the diverse university environment. Academic adjustment described variance in performance beyond the academic grades at the college level, the most traditional measure of academic success at the university level this makes academic adjustment a key concept in examining academic performance, especially the change from college to higher educational level. Several empirical studies [11, 12] have shown evidence of the relationship between students’ academic adjustment and academic success.

In academic adjustment is significantly associated with academic success, well-being, psychological capital, and positive emotions that contributed to students’ capabilities of dealing challenges. Prior studies proposed that psychological capital plays a significant role in students’ academic adjustment [13–15]. Psychological capital is conceptualised as best resources of positive expression. It is a mental state with strong efficacy and positive behaviour [16]. Academic excellencies are considered as predictors and promoters of individuals’ academic achievements. And there is significant and strong correlation between academic achievements and psychological capital [17].

Considering university environment, a social diversity, complexity, emotional states, and consequences can be found within the educational process. Individuals received subjective social support from each other. Anxious and stressed individuals perceived biased cognitive and emotions. Social support mediated between these negative characteristics and social satisfaction significantly. It reduced anxiety, negative emotions, helplessness, and loneliness. While extensive literature argued that social support positively associated with psychological capital. This strengthens academic adjustment with academic life in domains such as academic adjustment and academic achievement, as previous studies have indicated that academic adjustment impacts academic success [18]. Studies demonstrating the mechanisms through which relationships exist between perceived social support, psychological capital and success outcomes, especially for university students, with limitations such as sampling, methodology, environment, and socio-culture differences [19]. Henceforth, the present study perceived social support and psychological capital as part of investigation the universities of Pakistan. Identify this with gaps prior to university level in Pakistan [20]. This study examined the impact of perceived social support and psychological capital to academic adjustment in university students as key components supporting the success outcomes of university students.

## Literature review

Psychological capital is an important variable that reflects a positive psychological state of development previously; it has also been abstracted in general within positive psychology and in the investigation of the positive behaviour of organizations. The impact of psychological capital has primarily been tested with wellbeing, production and satisfaction within management and tested at the organizational level among employees. Few studies have examined the relationship of perceived social support and psychological capital with students’ academic achievements [14, 21, 22] as these researches examined the psychological capital and academic achievement among business students, organizational sector and college students. In this research paper, we have included perceived social support and psychological capital as higher-order constructs to evaluate their combined effect on academic adjustment and outcomes of success of university students [23]. This study addressed the theoretical as well as practical gaps in knowledge by examining how academic adjustment plays an important role as a mediator in perceived social support, psychological capital and outcomes of success relationships among university students.

### Perceived psychological capital

Psychological capital is an important factor that represents the positive psychological development of individuals. Psychological capital has generally been conceptualized in the context of positive behaviour, emotions, and expression. Psychological capital has four dimensions as resilience, optimism, hope, and self-efficacy. These dimensions stated fours aspects of behavioural potential to bounce back and forth when faced with adversity and problems to succeed; an optimistic feature of progress both now and in the future. Maintain objectives and, if possible, to redirect paths toward successful objectives, if possible. And, trust to take on and make every effort necessary to carry out demanding tasks [23]. University students required abilities and adjustment to develop psychological capital to face massive challenges. Unexpectedly, however, the contribution of psychological capital to university students’ academic adjustment and academic success has been relatively scarce to date [24]. Students who saw themselves as autonomous and competent and had a higher sense of connection were on average more optimistic, hopeful, resilient and self-effective and had higher grades. As a result, increasing the positive feelings of students has proven to develop positive qualities at the core of psychological capital, strengthen students’ interest in education and enhance academic adjustment and success [21, 22].

### Perceived social support

Social support recognized as beneficial for social life in a crucial social setup. Yet a concrete and universal definition is not uniformed [25, 26]. It can be understood as source of information and emotional material that developed under social networking. Individuals utilize social support that available in their environment [27]. Globally, studies have shown that multiple social capital types influence better mental health, development, health behaviours and educational outcomes among students. Social support means the feeling of a person being cared for and loved and a member of a socially supportive network [28]. Social support can be categorized into two types, namely, perceived, and actual social. However, the impact of actual social support on the mental health of students is lower than that of perceived social support. Prior studies have demonstrated that perceived social support is a significant component of youth development in different domains, such as problem behaviours [23], facets of psychological well-being and educational success. Social support from friends and family members contributes to the adjustment of students to their daily lives. The existence and development of a system of social support is an effective strategy for students at university as a means of reducing acculturation stress [23, 24] The university experience is the source of social networking creation. It develops students’ academic adjustment to reduce acculturative stress and social anxiety.

### Academic adjustment and academic success

Academic adjust is an ability of successful interaction within the academic environment. It develops potential to cope academic complexity and diversity [29]. In simple words it’s an understanding between students and academic environment. Explicitly, academic adjustment has four aspects as motivation of learning, clarity in educational goals, engaging and exerting academic work, and satisfaction with academic environment [30]. In the process of academic adjustment, there is rich history of students’ academic success. A positive academic adjustment between students and academic process persists to upcoming semesters and academic achievements [5]. The related literature explored tat academic adjustment with other influential factors (motivation, performance, learning potential etc.) and academic success positively associated with each other. Students’ academic performance is the outcome of their studying process and their academic effort toward education. Academic adjustment of students in higher education and concluded that motivation and self-efficacy predict academic success.

## The current study

### Hypothesises of the study

During university life, students are more mature and practitioners. They develop relationships with their peers, university mates, teachers, and other fellows. Each relation plays important role to develop academic adjustment through personal and social contributions [31]. Academic adjustment has been considered as a mediating variable in this study between perceived social support, psychological capital, and academic success of university students’ relationships. Academic adjustment is a comparatively new concept in behavioural studies thus, few studies in developed contexts have considered academic adjustment as a construct [14, 27, 28] however, very limited studies are found in developing contexts with social support and psychological capital prior to academic success [27, 28]. There are limited investigation regarding academic adjustment as moderator between social support, psychological capital, and academic success [32]. Especially at university level, few empirical studies have been conducted. This study fills this gap through explaining the mediating role of academic adjustment comprising social support and psychological capital for better academic achievements. The following research hypotheses were developed to analysed aforementioned objective of this study:

- H1: Perceived social support is positively linked to the academic adjustment of university students.
- H2: Perceived social support was positively linked to the academic success of university students.
- H3: Psychological capital is positively linked to the academic adjustment of university students.
- H4: Psychological capital is positively linked to the academic success of university students.
- H5: Academic adjustment positively associated with academic success of university students.
- H6: Academic adjustment significantly moderates the relationship between perceived social support and academic success of university students.
- H7: Academic adjustment significantly moderates the relationship between psychological capital and academic success of university students.

**Fig. 1:**
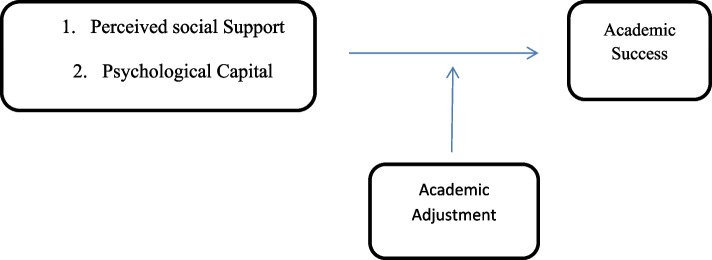
Conceptual framework of the research

Figure 1 showed that the hypothetical research framework indicates that the social support and psychological capital have direct relationship with academic success. The relationship among between these variables is likely to be moderated by academic adjustment.

### Significance of the study

The current study yields significant contribution for the body of knowledge. Firstly, it advances the academic literature by revealing that academic adjustment plays a moderating role in the complex interplay between perceived social support, psychological capital, and academic achievement within the higher education landscape. Secondly, it underscores the paramount importance of psychological capital in enhancing academic adjustment, emphasizing that the cultivation of psychological capital can effectively address the challenges faced by students, whether they stem from common factors or specific functional limitations. The research findings illuminate the direct and indirect influences of perceived social support and psychological capital on academic achievement. The outcomes underscore the importance of fostering an environment where students receive social support and can access psychological resources that bolster their commitment to academic pursuits. Students who perceive themselves as socially supported and psychologically equipped tend to navigate the academic demands of university life with greater ease. Consequently, this study provides the imperative for policymakers, educators, researchers, students, and education providers to proactively facilitate students’ effective adjustment to university life. Higher education institutions, in particular, are encouraged to prioritize the development of students’ academic skills, as such efforts are shown to significantly contribute to academic success and overall student well-being. Ultimately, the study's contributions extend beyond the realm of academia, offering valuable guidance for improving the overall quality of students’ educational journeys and their prospects for future success.

## Methods

A quantitative research method was adopted to test hypotheses. The variables in model aim to investigate the influential association of perceived social support and psychological capital factors on students’ academic success through academic adjustment. Self-reported data were collected from university students using a survey questionnaire. Participation was voluntary, and participants were asked to complete the questionnaires.

### Measures

#### Perceived social support

Perceived social support is a higher-order construct with three dimensions (i.e., friends, family and significant others). Each dimension contains four items each. The twelve items of perceived social support were adapted from Zimet, Dahlem [29]. The items of the scale were measured on a five-point Likert-type scale ranging from 1–5 “strongly disagree-strongly agree”. The sample items of each dimension were “I can talk about my problems with my family” (family), “My friends truly try to help me” (friends) and “I have a special person who is a real source of comfort to me” (significant others).

#### Psychological capital

Psychological capital is a higher-order construct with four dimensions (i.e., self-efficacy, resilience, hope, and optimism). Each subscale contains six items each. The 24 items of psychological capital were adapted from the study of Liran and Miller [7]. To measure psychological capital items, a five-point Likert-type scale was used ranging from 1–5 “strongly disagree-strongly agree”. The sample items of each subscale were “I feel confident analysing a study-related long-term problem to find a solution” (self-efficacy), “There are lots of ways around any study-related problem” (hope), “When things are uncertain for me as a student, I usually expect the best” (optimism) and “I can deal with study-related difficulties because I’ve experienced difficulty before” (resilience).

#### Academic adjustment

A total of six items for academic adjustment were adopted from Liran and Miller [7]. To measure academic adjustment items, a nine-point Likert-type scale was used ranging from 1–9 “suits me very much-does not suit me at all”. The sample item of the scale was “I find academic studies difficult”.

#### Academic success

We determined academic success as success outcome of students by average degree grades. Students CGPA of previous semesters was used as academic success. Students were asked to report their collective end of semester grades. In Pakistan higher education system, CGPA ranged from 1 to 4 and 2.5 is required to pass.

### Participants

A convenience sampling technique was adopted to select the sample for this study. The participants were enrolled in the two public universities in the Punjab province. There were 500 participants who were conveniently accessed by the researchers. All the students were undergraduates, graduates, and postgraduates in social sciences faculty. From which 203 were male students and 297 were female students. Most of the participants were between 20 and 30 years old. Because some participants are younger than 20 or older than 30 years old. A total of 500 questionnaires were distributed among the participants. The study yielded 384 questionnaires out of the total 500 distributed questionnaires with a response rate of 76.8%. Of the total received, 11 questionnaires were discarded due to their incompleteness; thus, 373 questionnaires were used for final data analysis. This decisively marked the final response rate to be 74.6%. Hence, the actual sample size of the study was 373 (Table 1).

**Table 1:** Demographics of the participants (*N* = 373)

	**Description**	**Frequency**	**Percentage**
Gender	Male	134	35.92
	Female	239	64.08
Marital Status	Married	56	15.01
	Unmarried	317	84.99
Age	Less than 20 years	61	16.35
	Between 21 and 25 years	207	55.50
	Between 26 and 30 years	90	24.13
	Above 30 years	15	4.02
Qualification	Undergraduate	187	50.13
	Graduate	146	39.14
	Postgraduate	34	9.12
	Others	6	1.61

### Procedure

All the data were collected through survey-based investigation process. The participants were briefed that their responses will be highly confidential. They can rate against statements voluntary. The participants expressed their perception, and experiences through self-reporting. After collection the data were organised and coded for statistical analysis. We applied descriptive and inferential statistical techniques through SPSS V.23, and SEM PLS. Frequencies, percentages, mean, standard deviation, structural equation modelling, correlation, and moderation were tested to verify the hypotheses.

## Results

### Assessment of reflective measurement model

The measurement model assessment involves examining individual item reliability (outer loading), internal consistency reliability, convergent validity (average variance extract) and discriminant validity (heterotrait-monotrait ratio of correlations) [5, 30, 31]. These instructions of measurement (outer) models were performed and interpreted below.

Table 2 results indicated that item loadings are between 0.619–0.870, which are within the range of 0.40 and 0.70. Similarly, the AVE of all latent constructs was between 0.550–0.623, which is above the threshold value of 0.50. Likewise, the composite reliability values of all latent constructs were between 0.840–0.892 and were above the threshold value of 0.70, as suggested by Hair, Hult [5]. However, the model produced variance inflation factor (VIF) values of LOC between 1.459 and 2.624, which were lower than the threshold value of 3 [32].

**Table 2:** Loadings, composite reliability, average variance extracted, VIF

**Higher Order Constructs**	**Lower Order Components**	**Items**	**Loadings**	**CR**	**AVE**	**VIF**
Psychological Capital	Self-efficacy	SE_1 SE_2 SE_3 SE_4 SE_5 SE_6	0.855 0.786 0.782 0.818 0.765 0.810	0.890	0.613	2.624 2.078 1.960 2.341 1.823 2.309
	Hope	H_1 H_2 H_3 H_4 H_5 H_6	0.690 0.711 0.789 0.838 0.790 0.763	0.840	0.560	1.493 1.604 2.114 2.462 1.428 2.102
	Resilience	R_1 R_2 R_3 R_4 R_5 R_6	0.753 0.696 0.750 0.806 0.778 0.839	0.863	0.595	1.544 1.979 2.492 1.878 2.322 2.166
	Optimism	OP_1 OP_2 OP_3 OP_4 OP_5 OP_6	0.825 0.760 0.730 0.831 0.867 0.766	0.885	0.622	1.797 2.630 2.160 2.375 2.480 2.267
Perceived Social Support	Family	FA_1 FA_2 FA_3 FA_4	0.870 0.826 0.792 0.865	0.892	0.623	2.463 2.341 2.281 2.573
	Friends	FR_1 FR_2 FR_3 FR_4	0.832 0.811 0.838 0.810	0.866	0.578	2.012 1.999 2.000 2.322
	Significant Others	SO_1 SO_2 SO_3 SO_4	0.794 0.850 0.848 0.761	0.851	0.602	1.939 2.311 2.196 2.471
Academic Adjustment		AA_1 AA_2 AA_3 AA_4 AA_5 AA_6	0.767 0.619 0.728 0.697 0.805 0.736	0.869	0.550	1.982 1.459 1.797 1.572 2.147 1.649

*SE* Self-efficacy, *H* Hope, *R* Resilience, *OP* Optimism, *FA* Family, *FR* Friends, *SO* Significant others, *AA* Academic adjustment

Discriminant validity is defined as “the extent to which a construct is truly distinct from other constructs by empirical standards” [5]. Table 3 indicates that all values were less than the threshold value of 0.85. This result indicates that the model has successfully achieved the construct’s discriminant validity.

**Table 3:** Discriminant validity

**Construct**	**AA**	**FA**	**FR**	**H**	**OP**	**OOS**	**R**	**SE**	**SO**
AA									
FA	0.824								
FR	0.722	0.636							
H	0.816	0.675	0.564						
OP	0.791	0.707	0.588	0.832					
OOS	0.829	0.764	0.705	0.773	0.75				
R	0.814	0.619	0.584	0.775	0.725	0.725			
SE	0.773	0.669	0.383	0.776	0.761	0.691	0.645		
SO	0.834	0.774	0.659	0.711	0.667	0.758	0.568	0.631	

*AA* Academic adjustment, *FA* Family, *FR* Friends, *H* Hope, *OP* Optimism, *OOS* Outcomes of Success, *R* Resilience, *SE* Self-efficacy, *SO* Significant others

### Structural model (direct and indirect effect)

In the structural model path coefficient assessment, there were seven hypotheses in which first five hypotheses had a direct effect and two had an indirect effect (through the mediating variable). The structural model evaluation showed path coefficients, standard error, t values and p values for the hypothesized relationships of the current study.

The results from Table 4 outlined that perceived social support has a positive and significant impact on academic adjustment (β = 0.466, t = 3.887, *p* < 0.05), hence supporting hypothesis 1. Furthermore, perceived social support also showed a significant and positive impact on outcomes of success (β = 0.306, t = 1.946, *p* < 0.05), therefore supporting hypothesis 2. However, psychological capital has a positive and significant relationship with academic adjustment (β = 0.480, t = 4.380, *p* < 0.05), thus supporting hypothesis 3. Moreover, psychological capital has a positive and insignificant relationship with outcomes of success (β = 0.245, t = 1.352, *p* > 0.05); therefore, hypothesis 4 was rejected. Similarly, the results from Table 5 indicate that academic adjustment has a positive and significant impact on outcomes of success (β = 0.335, t = 1.936, *p* < 0.05), therefore supporting hypothesis 5.

**Table 4:** Assessment of structural model direct relationships

**H**	**Direct Path Relationships**	**β-value**	**S.E**	***p*-value**	**Decision**
H1	Perceived social support—> Academic adjustment	0.466	0.120	.000	Supported
H2	Perceived social support—> Outcomes of success	0.306	0.157	.000	Supported
H3	Psychological capital—> Academic adjustment	0.480	0.110	.000	Supported
H4	psychological capital—> Outcomes of success	0.245	0.182	.061	Not-Supported
H5	Academic adjustment—> Outcomes of success	0.335	0.173	.000	Supported

**Table 5:** Moderating role of academic adjustment between social support, psychological capital, and academic success

**H**	**Indirect Paths Relationships**	**β-values**	**S.E**	***p*-values**	**Decision**
H6	Perceived social support -> Academic adjustment -> Outcomes of success	0.326	0.034	.001	Supported
H7	Psychological capital -> Academic adjustment -> Outcomes of success	0.211	0.079	.000	Supported

In Table 5, the results show that academic adjustment positively moderates the relationship between perceived social support and outcomes of success (β = 0.326, t = 1.660, *p* < 0.001), thus supporting hypothesis 6. Moreover, an indirect effect of psychological capital on outcomes of success has been tested, and we found that in terms of psychological capital, academic adjustment positively moderates the relationship between psychological capital and outcomes of success (β = 0.211, t = 1.807, *p* < 0.000), thus supporting hypothesis 7. The findings conclude that psychological capital affects university success outcomes through academic adjustment. The findings are in line with van Rooij and Jansen [11] conclude that psychological capital influences outcomes of success through academic adjustment.

### Variance explained and redundancy analysis

For the evaluation of the structural path model, the coefficient of determination is the most important criterion to be used for all endogenous latent constructs, commonly referred to as the determination of R^2^ [5, 31]. The coefficient of determination (R^2^) values show the variance percentage to show how much total variances in endogenous constructs could be explained by its independent constructs. A value of 0.19 was considered weak, 0.33 was considered moderate and 0.67 was considered substantial when using the PLS-SEM path modelling approach. Moreover, this study also uses Stone-Geisser’s method to examine the value of the predictive relevance (Q^2^) of the study model by using blindfolding. A cross-validated redundancy measure can be utilized to evaluate the predictive quality of the model, signified as Q2 [5].

Table 6 shows the total variances of two dependent latent constructs (i.e., academic achievement and outcomes of success). The study findings indicated that both perceived social support and psychological capital (independent variables) along with academic adjustment (moderator) explained 67.9% of the variance in outcomes of success (dependent variable). Moreover, both perceived social support and psychological capital together contributed to explaining 77.6% of the variance in academic adjustment. Table 6 also shows that the cross-validation redundancy (Q2) for academic achievement and outcomes of success are 0.374 and 0.486, respectively, which are far above zero, indicating the model’s predictive relevance.

**Table 6:** Variance explained and construct cross-validated redundancy analysis

**Latent Constructs**	**Variance Explained (R**^**2**^**)**		
Academic adjustment	77.6%		
Outcomes of Success	67.9%		
	**SSO**	**SSE**	**Q**^**2**^** (= 1-SSE/SSO)**
Academic adjustment	350.000	218.961	0.374
Outcomes of Success	150.000	77.068	0.486

## Discussion

The result of the path coefficient indicated that perceived social support had a positive and significant impact on academic adjustment. Thus, hypothesis 1 was supported. This suggests that university students can depend on social networks and their newly joined institutions as major support sources. The significance of social support is clearly demonstrated in a past study [14, 33]. Further demonstrates that perceived social support through social interactions is important [28, 34] and higher educational institutions for adjustment [27]. Likewise, the result of the path coefficient indicated a positive correlation between perceived social support and success outcomes. The result was therefore supported by hypothesis 3. The results of the study are consistent with those of past studies [28, 33]. Perceived social support can enable university students to strengthen their self-esteem and develop a positive social group that can provide university students with assistance and more support meet their basic needs, and successfully improve their success outcomes. Henceforth, social support from friends, family and significant others could have a major impact on university students’ success outcomes.

Similarly, the result of the path coefficient indicated that psychological capital was positively correlated with academic adjustment. The results of the SEM analysis fully confirmed hypothesis 3 and suggested that psychological capital, as a holistic resource, played an important role in the academic adjustment of students. The findings of this study are consistent with past studies showing that psychological capital has a significant impact on the academic adjustment of students [6, 35]. This implies that students’ confidence in their own psychological resources and skills is critical for successful academic adjustment and their capacity to recruit these skills in the face of well-defined goals and work for them with a positive attitude even when they face serious issues. In addition, the result of the path coefficient has shown a positive but insignificant link between psychological capital and success outcomes. Therefore, hypothesis 4 was not supported. In our context, this implies that such an endeavour does not pinpoint the potential of psychological capital in increasing students’ academic success while addressing the realities and requirements of academics. A plausible reason for the insignificant link between psychological capital and academic success could be that psychological capital does not have positive feedback on the academic success of university students. Henceforth, university students with limited psychological capital resources will no longer be able to lead learning processes that will ultimately reduce their academic success. The findings of this study contradict those of, [26], who all conclude that psychological capital is a significant predictor of students’ success outcomes.

Furthermore, the path coefficient result revealed a positive relationship between academic adjustment and success outcomes, confirming our fifth hypothesis [13]. This highlights that academic adjustment is significantly correlated with academic success, especially for students of higher education institutions who have a good balance between pre-university background attributes, friends and family support and the potential to deal with a wide range of adversities [32].

The outcomes of this study not only validate the hypotheses put forth but also provide empirical confirmation of the critical role that social support, particularly perceived social support, plays in shaping students’ experiences and outcomes in higher education such as the studies of Mushtaque et al. [36], Mishra [29], Raza, Qazi, and Yousufi [6], and Hazan Liran and Miller [18]. These results not only build upon the existing literature but also strengthen our understanding of the intricate interplay between social support and students’ adjustment and success in university settings [5]. The findings of this study align with and contribute to the existing body of research on the significance of social support, specifically perceived social support, in the context of higher education [37]. These results underscore the vital role that social networks and support systems play in students’ experiences and outcomes during their university journey.

Contradictory evidence in the literature would involve studies that challenge the notion of social support as a uniformly positive factor in students' academic lives [16]. For example, some research may suggest that excessive reliance on social support networks can potentially hinder students’ development of self-reliance and problem-solving skills, thus impeding their overall growth and adaptation to university life [29, 36]. Such studies might argue that an overreliance on perceived social support might not always be beneficial. Similar references in the literature would include studies that have consistently highlighted the positive impact of social support on students’ well-being, academic performance, and adjustment to university life [18]. These studies would support the idea that social support networks, including family, friends, and institutions, contribute significantly to students’ overall success and satisfaction in higher education [6, 29, 36, 37]. In essence, while there may be some contradictory perspectives in the literature, the prevailing body of research tends to align with the findings of this study, emphasizing the pivotal role of perceived social support in shaping students’ experiences and outcomes in higher education [18].

A novel study finding is the moderating effect of academic adjustment within these relationships. More particularly, we observed support for our hypotheses that the link between psychological capital, perceived social support and academic achievement would be moderated by academic adjustment [27]. The results demonstrated that psychological capital and perceived social support positively influence academic outcomes through academic adjustment [29] The results of the study outlined that academic adjustment partially moderated the relationship between perceived social support and the academic achievement of university students. Furthermore, the findings also revealed that academic adjustment fully moderated the relationship between the psychological capital and academic achievement of university students [11, 35]. The results supported hypotheses 6 and 7 of this study. Thus, the higher the level of university students’ perceived social support and psychological capital were, the higher the degree of their academic adjustment. Accordingly, they would have a greater degree of academic success outcomes. These results highlight the significance of academic adjustment as a strong precursor of academic performance. It is evident that increasing psychological capital is more feasible as perceived social support rises [37]. Students will have increased psychological capital, indomitable tenacity, and pressure-handling confidence. They will be more motivated to improve themselves, exert great effort in the correct direction, remain positive and optimistic in the face of adversity, and grow positively and healthily. This demonstrates the importance of parent-school cooperation in child development. Teachers and parents must assist junior high school students develop the ability to recognize social support, in the sense of a peaceful atmosphere of support, and fully utilize their own positive psychological quality in order to establish a solid foundation for their healthy development [36]. This indicates that psychological capital and social support resources influence academic adjustment, which in turn influences academic success.

## Research implications

The findings of the current study contribute to the academic literature in several ways. First, one of the contributions made by the present study is primarily the finding that academic adjustment moderates the relationships between perceived social support, psychological capital and academic achievement in the context of higher education. Second, psychological capital was found to have a greater influence on academic adjustment. This implies that the role of psychological capital in improving academic adjustment cannot be overlooked and that its development seriously addresses the challenges faced by students, whether they are common to all students or because of certain functional detriments. The results indicate that perceived social support and psychological capital have direct as well as indirect impacts on outcomes of success.

This research has practical implications as well. This result allows all parents, teachers and departments to improve student engagement and inspire them to study regularly. Students who feel socially motivated and psychologically resourced to study can easily cope with the academic demands of the university. The current study therefore indicates that it is necessary for policy makers to make full use of their ability to enable students to adjust to university life effectively. Higher education institutions should therefore pay full attention to the development of students’ academic skills that contribute to academic success.

## Limitations and future research directions

The fact that academic adjustment was the only moderator included in the study is the most significant limitation, considering that it is the most significant correlate of achievement. As a result, it would be beneficial for future research to cover not just the personal-emotional and social aspects of adjustment but also the institutional aspects. This study is a basic moderational study that indicated partial moderation between the two constructs; however, future research could investigate the correlation with multi-moderation studies that include more than one variable that moderates the relationship between the two constructs. The sample for this study was recruited from just a couple colleges in the province of Punjab in Pakistan, which is a limitation. These results could be replicated in future studies by expanding the scope of their investigation to include more nations, regions, and academic disciplines. All variables were measured at a single point in time, making it difficult to identify causal relationships. Therefore, to determine causal relationships, future studies should conduct longitudinal research.

## Conclusion

This research explores whether perceived social support and psychological capital affect students’ academic adjustment and outcomes of success and the impact of adjustment on success outcomes. According to the findings, students who reported receiving social support while obtaining academic information had greater levels of academic adjustment. This indicates that these students were better prepared to deal with the challenges that were presented by the rigorous educational environment. According to correlation and structural equation modelling (SEM) research, it has been found that when students are confronted with academic problems, they are more likely to draw from positive resources, such as psychological capital, that contribute to greater academic adjustment. According to the social capital theory and the conservation of resources theory, which were utilized in this research, successful contact with the academic environment and engagement in academic activities leads to academic adjustment and academic achievement. These hypotheses are confirmed by the findings of the study, which provide credence to the findings. Students at universities who have successfully navigated the academic adjustment process are more likely to have high cumulative grade point averages (CGPAs) and to be on track to get their degrees. The findings indicate that academic adjustment to the environment of the university is helpful for predicting positive connections between psychological capital and perceived social support constructs.

## Data Availability

The datasets used and/or analyzed during the current study are available from the first author upon reasonable request.
